# Mechanism of Metal Complexes in Alzheimer’s Disease

**DOI:** 10.3390/ijms252211873

**Published:** 2024-11-05

**Authors:** Yi Liu, Jiaying Ma, Qianling Zhang, Yi Wang, Qi Sun

**Affiliations:** 1 Guangdong Key Laboratory for Genome Stability & Disease Prevention, International Cancer Center, Department of Pharmacology, Shenzhen University Medical School, Shenzhen University, Shenzhen 518055, China; 2021221095@email.szu.edu.cn (Y.L.); 2021221071@email.szu.edu.cn (J.M.); 2 College of Chemistry and Environmental Engineering, Shenzhen University, Shenzhen 518055, China; zhql@szu.edu.cn

**Keywords:** metal complexes, mechanism, Alzheimer’s disease, therapeutics

## Abstract

Alzheimer’s disease (AD) is a kind of neurodegenerative diseases characterized by beta-amyloid deposition and neurofibrillary tangles and is also the main cause of dementia. According to statistics, the incidence of AD is constantly increasing, bringing a great burden to individuals and society. Nonetheless, there is no cure for AD, and the available drugs are very limited apart from cholinesterase inhibitors and N-Methyl-D-aspartic acid (NMDA) antagonists, which merely alleviate symptoms without delaying the progression of the disease. Therefore, there is an urgent need to develop a medicine that can delay the progression of AD or cure it. In recent years, increasing evidence suggests that metal complexes have the enormous potential to treat AD through inhibiting the aggregation and cytotoxicity of Aβ, interfering with the congregation and hyperphosphorylation of tau, regulating dysfunctional synaptic and unbalanced neurotransmitters, etc. In this review, we summarize the current metal complexes and their mechanisms of action for treating AD, including ruthenium, platinum, zinc, vanadium, copper, magnesium, and other complexes.

## 1. Introduction

**Figure 1:**
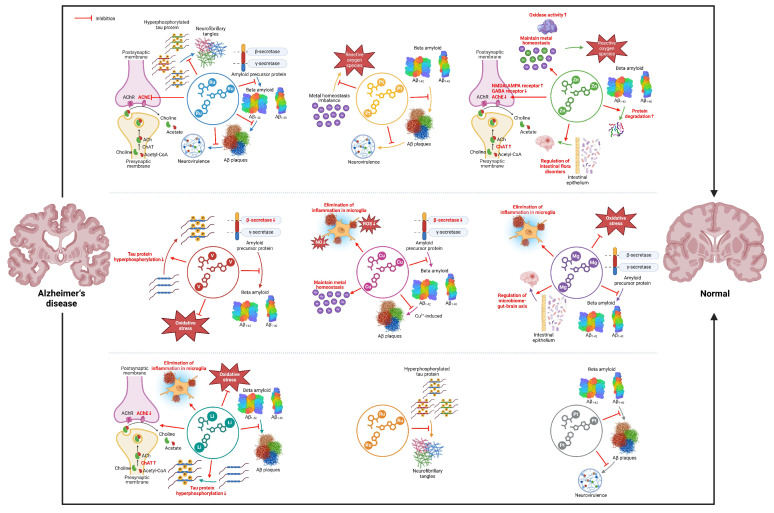
Major mechanisms of action of metal complexes in AD.

**Figure 2:**
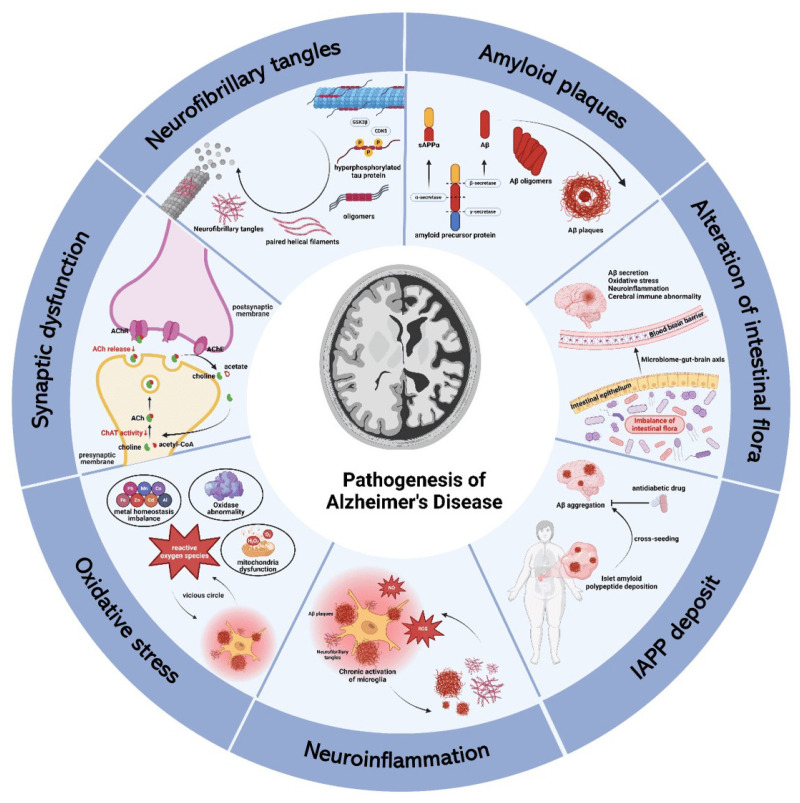
The pathogenesis of Alzheimer’s disease.

The research into AD originated from Alois Alzheimer’s autopsy of a 51-year-old woman in 1907, in which he reported that the woman had exhibited rapid sexual abnormalities and cognitive dysfunction [1]. Nowadays, AD is considered to be a chronic neurodegenerative disease, and its prevalence will increase dramatically as the population ages [2]. According to reports published by the Alzheimer’s Association in 2023 and 2024, there are 6.7 and 6.9 million, respectively, Americans age 65 and older living with AD, and the number could grow to 13.8 million by 2060, barring the development of medical breakthroughs to prevent, slow, or cure AD [3,4]. The disease is associated with a variety of causes, such as age, gender, genetic factors (*APOE ϵ 4 allele, APP gene*, etc.), head injury, vascular disease, infection, and environmental factors [5,6,7,8]. These causes together lead to the characteristic lesions of Alzheimer’s patients: the formation of senile plaques and neurofibrillary tangles that cause irreversible neurodegeneration, resulting in cognitive impairments such as apraxia, aphasia, and agnosia, and most patients can simultaneously develop neuropsychiatric symptoms such as apathy, depression, aggression, anxiety, and sleep disorders, eventually leading to death [8,9,10,11]. In addition to the impact on individuals, the disease also imposes a serious burden on society, with the United States spending 360 billion dollars in 2024 on Alzheimer’s patients aged 65 years and older [4]. Therefore, the prevention and treatment of AD have become a hot spot, but there is still no cure, mainly symptomatic treatment to delay the progress of the disease [12]. Current treatments include cholinesterase inhibitors (such as galantamine, donepezil, and cabalatine) and NMDA antagonists (such as memantine), which improve memory and alertness but do not stop the progression of disease [13]. In addition to immunotherapy, there is gene therapy, probiotic therapy, and diet therapy [14,15,16,17]. At present, more and more evidence shows that the change in metal homeostasis is an important factor in the pathogenesis of AD [18,19,20]. Metal complexes are considered to be potential therapeutic agents for AD [21,22]. In recent years, the research and development of metal complexes are also maturing, which have been proven to have antitumor, antibacterial, antioxidant, and CT imaging effects [23]. This review focuses on the progress that mechanisms of metal complexes have in treating AD and briefly describes the pathogenesis of AD (Figure 1 and Figure 2).

## 2. The Pathogenesis of AD

### 2.1. Amyloid Cascade Hypothesis

The deposition of β-amyloid (Aβ) and the formation of senile plaques are the main factors leading to the onset of AD, and ther are also among the most important pathological features of AD [24]. Under pathological conditions, Aβ is the proteolytic product of the amyloid precursor protein (APP) produced by β-secretase (BACE1) and γ-secretase through the amyloidosis pathway. Under physiological conditions, APP is a soluble APPα fragment (sAPPα) catalyzed by α-secretase rather than β-secretase via a non-amyloid pathway [25]. The deposition of Aβ is the result of various factors, including genetics, molecular, and cytology. Mutations in several genes, such as *apolipoprotein ϵ 4 (APOE ϵ 4), APP, presenilin 1 (PSEN1), and presenilin 2 (PSEN2)*, affect Aβ metabolism and rapidly lead to Aβ accumulation [25,26,27]. *APOE ϵ 4 and PSEN* are important risk factors for sporadic/delayed-onset and familial/early-onset AD, respectively. The former promotes the deposition of Aβ by decreasing the clearance of Aβ, and the latter promotes the synthesis of Aβ by increasing the activity of γ-secretase [25]. In addition, the expression of low-density lipoprotein receptor-associated protein 1 (LRP1) and P-glycoprotein (P-gp/ABCB1), the transporters that mediate Aβ outflow from the blood–brain barrier (BBB), are decreased in AD patients, and inversely, the expression of the receptor of advanced glycation end products (RAGE), a transporter that mediates the inflow of BBB, is increased, ultimately leading to Aβ deposition [28,29,30]. At the same time, the BBB of AD patients is destroyed, the expression of ATP-dependent P-gp is decreased, and the Aβ cannot be transferred from cell to cell, thus affecting the clearance of Aβ [31,32,33]. In addition, metal ions such as copper, iron, zinc, and aluminum can promote the accumulation and deposition of Aβ by binding with Aβ [34,35]. Deposited Aβ can bind to a variety of receptors to induce mitochondrial dysfunction and oxidative stress in AD neurons, resulting in a large amount of calcium ions flowing into neurons and causing neurotoxicity [36]. In addition, the degeneration of cholinergic neurons caused by the neurotoxicity of Aβ can further cause cognitive decline and memory loss [5].

### 2.2. Neurofibrillary Tangles

Neurofibrillary tangles (NFTs) are another important pathological feature of AD. NFTs consist of the microtubule-associated protein tau, which aggregates in pairs in a spiral filamentary pattern [37]. Under physiological conditions, the tau protein interacts with tubulin heterodimers in the axon segment of the neuronal cytoskeleton, thereby promoting the polymerization of tubulin into microtubules and stabilizing the microtubules [38]. Microtubules are the main structure of the cellular skeleton of neurons and play an important role in the function and development of neurons. In AD, tau kinases such as glycogen synthase kinase 3 β (GSK3β), cyclin-dependent kinase 5 (CDK5), and caspase are activated, resulting in tau protein hyperphosphorylation and polymerization into insoluble NFTs. However, insoluble NFTs can lead to axon transport dysfunction, mitochondrial and cytoskeletal dysfunction, the abnormal activation of microglia, and local neuroinflammatory response [25,39]. In addition, the activation of caspase in AD patients causes in situ carboxyl and amino amputation of the tau protein, and the truncated form of the tau protein is also neurotoxic [37].

### 2.3. Synaptic Dysfunction and Neurotransmitter Imbalance

Synaptic dysfunction is one of the causes of cognitive dysfunction in AD patients. Synaptic dysfunction is mainly manifested by abnormal changes in synaptic plasticity. Synaptic plasticity refers to the establishment and strength of connections between neurons, mainly including short-term synaptic plasticity and long-term synaptic plasticity, which is further divided into long-term potentiation (LTP) and long-term depression (LTD). AD patients often exhibit the abnormal inhibition of LTP and the enhancement of LTD [25]. Under physiological conditions, Aβ plays a neuroregulatory role by controlling synaptic activity and neurotransmitter release from presynaptic endings, and abnormally elevated soluble Aβ in AD patients can induce synaptic dysfunction, disturb the excitation/inhibition balance of neural circuits, and then alter normal neural network activity, leading to cognitive decline [40,41,42]. In addition, Jens V Andersen et al. reported that in the 5xFAD mouse model of AD, the dysmetabolism of astrocytes leads to inadequate glutamine synthesis, which directly impedes neuronal γ-aminobutyric acid (GABA) synthesis, which may underpin synaptic dysfunction and neurotransmitter imbalance in AD [43].

Among the neurotransmission systems of AD imbalance, the cholinergic system has been studied deeply at present. Acetylcholine is one of the most important neurotransmitters of the brain, and it is involved in many basic functions such as promoting synaptic plasticity and neural network connectivity, synchronizing neuronal activity, and increasing cerebral blood flow. The AD cholinergic hypothesis is based on three main pieces of evidence: (1) the depletion of presynaptic cholinergic markers in the cerebral cortex of AD patients, (2) severe degradation of the nucleus basalis of Meynert (NBM) as a source of cortical cholinergic energy, and (3) cholinergic agonists, which can enhance memory in AD patients. In addition to reduced sources of cholinergic neurotransmitters, AD also manifests as abnormalities in the number and function of cholinergic receptors [44]. Based on the cholinergic hypothesis, three acetylcholinesterase inhibitors, including donepezil, rivastigmine, and galantamine, have been approved by the U.S. Food and Drug Administration (FDA) for the treatment of AD [25].

Apart from the cholinergic system, there are also neurotransmitter systems such as the glutamatergic system, GABA ergic system, and the monoaminergic system that are involved in the occurrence and development of AD. For example, synaptic loss and glutaminergic dysfunction were observed in patients with AD, and α-amino-3-hydroxy-5-methyl-4-isoxazolepropionic acid (AMPA) receptors and NMDA receptors were downregulated in the hippocampus [25]. GABA is an important inhibitory neurotransmitter that plays an important role in balancing brain excitability and activity. Less GABA expression was detected in both normal and animal models [45,46]. Normal astrocytes do not produce the inhibitory neuroglial transmitter GABA, and Seonmi Jo et al. found that reactive astrocytes abnormally produce and release a large amount of GABA in the AD mouse model, while inhibiting GABA to produce and release astrocytes can completely restore the damage caused by it. In addition, Yuji Yoshiike et al. found that the GABA (A) receptor antagonist bicuculline can repair cognitive deficits in a pathological mouse model of AD. Monoamine neurotransmitters include catecholamines (CA), which include dopamine (DA), norepinephrine (NA), epinephrine, and indoleamines, which are mainly 5-hydroxytryptamine (5-HT). Studies have shown reduced expression of DA receptors in the brains of AD patients [47,48]. Several studies have found altered expression of dopaminergic neuronal markers such as D2-like receptors, dopaminergic transporters, and tyrosine hydroxylase in the nucleus accumbens (NAcc) of the AD brain, and imaging tests suggest atrophy in this region [49,50,51,52]. α2A adrenergic receptors regulate APP endocytosis and promote Aβ production by disrupting the interaction between APP and the vacuolar protein sorting 10 (Vps10) family of proteins [53]. NA has a neuroprotective effect, whose concentration in the locus coeruleus (LC) salient region is reduced after exposure to Aβ, promoting the inflammatory response of microglia, thereby impacting the migration and phagocytosis functions of microglia and ultimately reducing the clearance of Aβ [54]. Postmortem reports of AD patients suggest reduced levels of 5-HT and its receptor [55]. The disorder of the 5-HT system is closely related to the occurrence of depression, and studies have found that the risk of AD in depressed patients is significantly increased [56]. In addition, the antidepressant fluoxetine is a class of selective 5-HT reuptake inhibitors, and study results suggest that it can improve spatial learning and cognitive ability in patients, as well as reduce plasma Aβ content in patients [57,58].

### 2.4. Oxidative Stress

Oxidative stress is regarded as one of the crucial factors in the early onset of AD, and it has a reciprocal causal relationship with hypotheses such as the amyloid cascade reaction, NFTs, and neuroinflammation [59]. Oxidative stress can facilitate the aggregation of Aβ by influencing the expression of *APP* and regulating the activity of its associated secretase enzymes. Renren Bai et al. discovered that when human neuroblastoma cells were placed in a pro-oxidative environment, the expressions of γ-secretase, BACE1, and PSEN increased, while the expression of α-secretase decreased, and the sAPPα produced through the non-amyloidogenic pathway reduced [60]. This indicates that oxidative stress might promote the abnormal processing of APP in human neuroblastoma cells. Oxidative stress can also result in tau hyperphosphorylation and the formation of NFTs. When the normal function of tau is interfered with by H_2_O_2_ and reactive oxygen species (ROS), it interacts with CDK5 and GSK3β kinases, causing tau hyperphosphorylation. The hyperphosphorylation and aggregation of the tau protein, in turn, promote the generation of ROS. Bo Su et al. demonstrated through chronic oxidative stress neuronal cell models that chronic oxidative stress leads to increased tau phosphorylation and aggregation, playing a critical role in the formation of cerebral neurofibrillary pathology [61]. In the neuroinflammation hypothesis, microglia are one of the main sources of pro-inflammatory factors. The stimulation of ROS further activates them, releasing a series of inflammatory factors and in turn promoting the release of ROS. Moreover, oxidative stress in neurons can lead to mitochondrial dysfunction, DNA damage, and epigenetic changes associated with cognitive aging and AD, further promoting disease progression [62]. In summary, oxidative stress can promote the aggregation of Aβ, the phosphorylation of tau, and the inflammatory response of the nerves, and these secondary pathological reactions will promote redox imbalance and eventually form a vicious cycle [63]. Additionally, the occurrence of oxidative stress is considered to be closely related to metal homeostasis imbalance, abnormalities in related oxidases, mitochondrial homeostasis imbalance, and the generation of excessive ROS [59]. It can damage neuronal membrane lipids, proteins, and nucleic acids, ultimately leading to neuronal cell death [64]. Based on the significant position of oxidative stress in the pathogenesis of AD, current studies are exploring the possibility of various antioxidant therapeutic strategies for the treatment of AD. The research conducted by Maria Calvo-Rodriguez et al. revealed that the aggregation of soluble Aβ oligomers results in an elevated level of mitochondrial oxidative stress. Moreover, inhibiting mitochondrial Ca^2+^ influx or applying the mitochondria-targeted antioxidant SS31 (also known as Elamipretide) can rectify the oxidative stress driven by Aβ plaques and ameliorate Aβ plaque-related dystrophic neurites [65].

### 2.5. Neuroinflammation

Microglia are among the major immune cells in the brain. Based on proteomic and cytomic analyses, microglia can be classified into disease-associated microglia (DAM), microglial neurodegenerative phenotype (MGnD), morphologically activated microglia (PAM), and other unnamed subgroups [66,67,68,69]. The aggregated Aβ in the early stage of AD onset activates microglia, causing them to transform into the DAM subtype and exert neuroprotective effects by phagocytosis and proteolysis to clear Aβ. However, when Aβ cannot be completely removed, microglia will be chronically activated and undergo inflammatory reactions. Subsequently, by promoting the expression of *APP* and increasing the release of iron, this further promotes the aggregation of Aβ. Additionally, the aggregation of tau further intensifies the activation of microglia, promoting the generation and release of inflammatory factors, including ROS and nitric oxide (NO). These inflammatory factors not only amplify the immune response and cause neurotoxicity but also continuously trigger the activation of neuronal p38 mitogen-activated protein kinase (p38 MAPK) and further stimulate the hyperphosphorylation of tau, leading to the establishment of a vicious cycle [70,71,72,73].

### 2.6. Imbalance of Intestinal Flora

Infection, the mode of delivery, the use of antibiotic drugs, the nature of nutrient supply, and environmental stress can all contribute to intestinal flora disorders [74]. The imbalance of intestinal flora can affect the brain immune balance through the microbiome–gut–brain axis and play a key role in the pathogenesis of AD [75]. The dysregulation of intestinal flora can promote the secretion of lipopolysaccharide and amyloid protein, thus affecting intestinal permeability and BBB. In addition, dysregulation of the gut microbiota can promote oxidative stress, neuroinflammation, Aβ formation, insulin resistance, and ultimately nerve death [76].

### 2.7. Islet Amyloid Deposition

Islet Amyloid Polypeptide (IAPP) is an endocrine peptide hormone consisting of 37 amino acids and four functional structural regions. IAPP is secreted with insulin in pancreatic β cells. The deposition of IAPP is considered to be an important pathological feature of type 2 diabetes mellitus [77]. There is a lot of evidence that IAPP deposition is closely related to the occurrence of AD. First, several fragments in IAPP have been shown to form amyloid fibrils, including short peptide fragments such as SNNFGAILSS, NFGAIL, and NFGAILSS, and the formation of amyloid fibrils is an important pathological feature of AD [78]. Statistically, the incidence of AD has increased in people with type 2 diabetes, and a series of studies have found that hypoglycemic drugs, including rosiglitazone, pioglitazone, and liraglutide, can reduce the deposition of Aβ or tau proteins in the body, thereby improving cognitive performance in patients [79,80,81,82,83]. In addition, anti-IAPP aggregation drugs may also reduce amyloid aggregation and improve cognitive function in AD patients [84,85,86]. Following the discovery and hypothesis of a significantly increased incidence of AD in patients with type 2 diabetes, I Moreno-Gonzalez et al. found that inoculation of IAPP aggregates in the brain of animals can promote Aβ aggregation through an interaction similar to “cross-seeding”, ultimately leading to cognitive and memory decline in animals [87]. The study of G. Zhang et al. directly proved that IAPP can promote tau protein aggregation, indicating that there is also a cross-seeding interaction between the IAPP and tau protein [88].

## 3. Progress of Various Metal Complexes in the Treatment of AD

### 3.1. Ruthenium Complex

**Figure 3:**
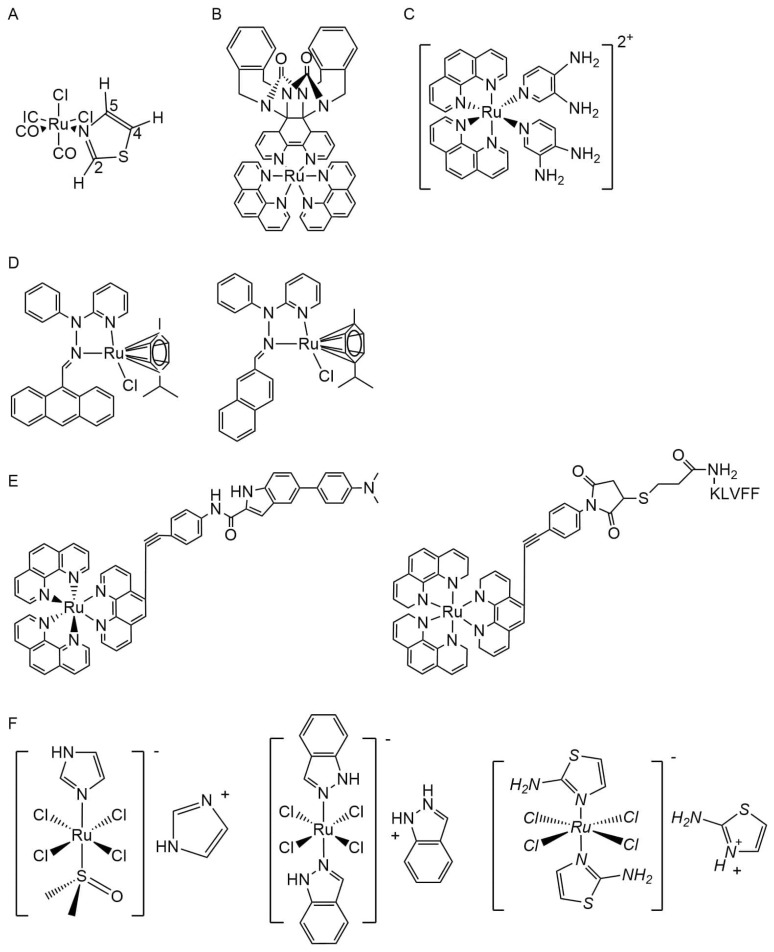

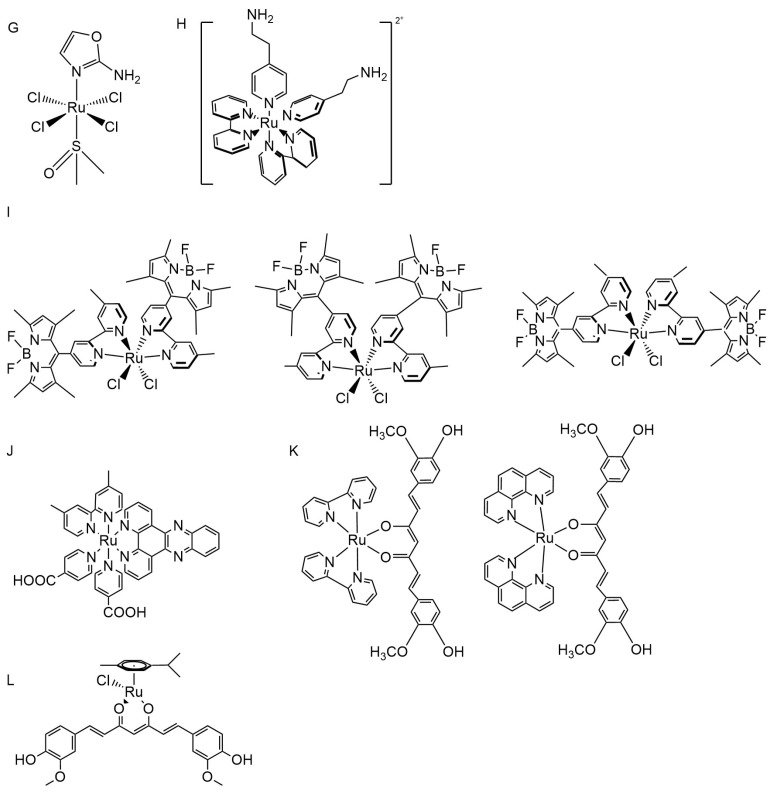
The chemical structures of ruthenium complexes. (**A**) *fac*-[Ru(CO)_3_Cl_2_(*N*^1^-thz)]; (**B**) [Ru(phen)_2_(bxbg)]^2+^; (**C**) RuApy (*cis*-[Ru(phen)_2_(3,4Apy)_2_]^2+^); (**D**) [Ru(p-cymene)Cl(L-1)][PF_6_] and [Ru(p-cymene)Cl(L-2)][PF_6_]; (**E**) Ru-WJ and Ru-YH; (**F**) NAMI-A, KP1019, PMRU20; (**G**) Oc(oxazolyl-based Ru(III) complexes); (**H**) [Ru(bpy)_2_(EtPy)_2_]^2+^; (**I**) BODIPY-ruthenium conjugates; (**J**) [Ru(dmbpy)(dcbpy)dppz)]; (**K**) Ru-1 and Ru-2 (Ru(II) complex scaffold with curcumin molecules); (**L**) Aromatic Ru(II) derivatives of curcumin.

The abnormal increase and aggregation of toxic Aβ to form plaques is considered to be the initial pathological change of AD, which is also one of the important targets of AD disease-modifying therapy (DMT). In AD, ruthenium complexes mainly inhibit Aβ protein aggregation and fibrous plaque formation. The idea for Ru complexes to treat AD came from the research of Daniela Valensin et al. in 2010, where they found that Ru(II) complex *fac*-[Ru(CO)_3_Cl_2_(*N*^1^-thz)] (I hereafter; thz = 1,3-thiazole) (Figure 3A) can selectively interact with Aβ_1–28_ amino acid residues to form a stable Ru-Aβ_1–28_ admixture, demonstrating that ruthenium complexes can be used as potential therapeutic agents for AD [89]. Due to the complexity and multifactor nature of Aβ, Nilima A. Vyas et al. designed and synthesized a Ru(II) polypyridine complex [Ru(phen)_2_(bxbg)]^2+^(phen = 1,10-phenanthroline, bxbg = bis(o-xylene)bipyridine glycoluril) (Figure 3B) that can target multiple targets, as well as can not only inhibit the activity of acetylcholinesterase but also inhibit the aggregation ability of Aβ [90]. S. Chan and his team synthesized a novel tetradentate Ru(II) complex [Ru(tpa)(bt)]ClO_4_(tpa = tris(2-pyridylmethyl)amine, bt = 2-acetylbenzo[b]thiophene-3-olate), which inhibits Aβ_1–40_ aggregation in vitro [91]. Mariana P. Cali et al. found that *cis*-[Ru(phen)_2_(3,4Apy)_2_]^2+^(phen = 1,10-phenanthroline, 3,4 Apy = 3,4-diaminopyridine) (Figure 3C) interferes with Aβ_1–40_ aggregation and protects PC12 nerve cells from the toxic damage of Aβ_1–40_ in the early aggregation stage, keeping the cells 100 percent active. Sain Singh et al. designed two novel Ru complexes [Ru(p-cymene)Cl(L-1)][PF_6_] and [Ru(p-cymene)Cl(L-2)][PF_6_] (Figure 3D), finding that both of them can bind to Aβ_1–42_ and significantly inhibit the formation of Aβ_1–42_ amyloid fibers; among them, [Ru(p-cymene)Cl(L-2)][PF_6_] also can protect PC12 neurons from Aβ_1–42_ cytotoxicity [92]. X. Chen and his team synthesized two ruthenium polypyridine complexes, Ru-WJ and Ru-YH (Figure 3E) (complex of Ru polypyridine with organic fluorophores (*N, N*-dimethylaniline) and hydrophobic peptides (KLVFF)), and they found that both of them could detect Aβ formation and had no obvious toxicity to normal cells and tumor cells. In addition, Ru-YH also had the function of inhibiting Aβ growth and fiber formation [93].

As reported by Michael R Jones and his team, KP1019 (Figure 3F) (indazolium *trans*-[tetrachlorobisindazole ruthenate(III)]) regulates Aβ aggregation, promotes the formation of soluble Aβ aggregates, and inhibits Aβ toxicity to SH-SY5Y human neuroblastoma cells [94]. According to Luigi Messori and his team, PMRU20 (Figure 3F) (2-aminothiazolium [*trans*-RuCl_4_(2-aminothiazole)_2_]) can antagonize the toxicity of Aβ_25–35_ and inhibit the toxicity of Aβ_1–42_ to neurons [95]. Gideon K. Yawson et al. found that oxazoly-based Ru(III) complexes (Figure 3G) not only inhibit the aggregation of Aβ_1–28_ but also decompose the formed amyloid aggregates [96].

Metal complexes can exert different biological activities by changing their subtle chemical structures, that is, producing different structure–activity relationships (SARs). Samantha E. Huffman et al. and Gideon K. Yawson et al. prepared thiazolyl derivatives of NAMI-A (Figure 3F) ([ImH][RuCl_4_(DMSO)(Im)], DMSO = dimethyl sulfoxide, Im = imidazole) and imidazolyl derivatives of PMRU20, respectively, to explore the SAR treatment of AD by different Ru(III) complexes. The former group found that functional groups in thiazole groups regulate Aβ aggregation, NH_2_ > CH_3_ > H, and the latter group found that imidazolyl complexes had a better anti-Aβ aggregation effect than thiazolyl complexes [97,98]. Brendan J. Wall et al. designed 11 NAMI-A pyridinyl derivatives using different functional groups to replace the fourth position of the pyridinium ring. The results showed that the NAMI-A derivatives had the strongest effect on Aβ aggregation and cytotoxicity when the amino group replaced the fourth position of the pyridinium ring [99]. Johanna T. Ehlbeck et al. constructed four Ru(III) complexes (RuP1-4) according to [(DMSO)_2_H][*trans*-Ru(DMSO)_2_Cl_4_] to explore the effect of the position of a primary amine in the pyridine group on the anti-amyloid activity of the complexes. All four complexes showed little cytotoxicity, among which RuP2 had the best ability to bind Aβ and regulate its aggregation. The cytotoxicity of RuP3 was the lowest due to the regulation of Aβ aggregation [100]. Sara La Manna and her team designed three diruthenium complexes [Ru_2_Cl(DPhF)(O_2_CCH_3_)_3_]·H_2_O, [Ru_2_Cl(DPhF)_2_(O_2_CCH_3_)_2_]·H_2_O, and K_2_[Ru_2_(DPhF)(CO_3_)_3_]·3H_2_O (DPhF = *N, N′*-diphenylformamidinate), where it was found that the first two Ruthenium complexes inhibited Aβ_1–42_ aggregation and reversed its cytotoxicity, while the third complex did not have this effect [101].

The reduction of cholinergic neurons is also one of the pathogenesis processes of AD, and cholinesterase inhibitors targeting this mechanism are currently among the few drugs approved for the treatment of Alzheimer’s disease, including donepezil, rivastigmine, galantamine, and huperzine A. Several studies have found that ruthenium complexes have cholinesterase inhibitory activity, which may be a new drug for the treatment of AD. The novel Ru(II.) complex [Ru(bpy)_2_(EtPy)_2_]^2+^ (bpy = 2,2′ bipyridine, EtPy = 4,2 Ethylamino Pyridine) developed by Marlon P. Almeida and his team has cholinesterase inhibitory activity similar to rivastigmine at low concentrations and [Ru(bpy)_2_(EtPy)_2_]^2+^ (Figure 3H) compared to bpy, and EtPy single ligands have a stronger interaction with cholinesterase [102]. Jerneja Kladnik and his team synthesized eight organic Ru(II) chlorides with pyrithione-type ligands, and the results show that this complex can inhibit the activity of cholinesterase, including the acetylcholinesterase (AChE) produced in the early stage of AD and the butyrylcholinesterase (BuChE), which continues to exert its activity later, but the ligand alone does not have this effect [103].

Ruthenium complexes can also be coupled with other substances to form more complex complexes that treat the effects of AD through a variety of pathways. C. Wu and his team designed and synthesized boron-dipyrromethene (BODIPY)-ruthenium conjugates (Figure 3I), in which BODIPY is a class of photosensitizers, and they found that this complex can promote neuron growth and increase its length and number. In addition, the photosensitizer dipyrromethene can also induce singlet oxygen generation, which is also thought to have anti-toxic Aβ aggregation activity [104]. Eththilu Babu et al. used the systematic evolution of ligands by exponential enrichment (SELEX) system to select suitable RNA or DNA molecules as aptamers and formed a complex with [Ru(dmbpy)(dcbpy)dppz)] (dmbpy = 4,4′-dimethyl-2,2′-bipyridine, dcbpy = 4,4′-dicorboxy-2,2′-bipyridine, dppz = dipyridophenazine) (Figure 3J), which has a stronger inhibitory effect on the formation and aggregation of Aβ fibers [105]. Many studies have shown that curcumin and its metal complexes can exert anti-AD activity through various mechanisms, such as blocking Aβ aggregation, chelating neurotoxic metal ions, and scavenging oxygen free radicals [106]. W. Liu and his team conjugated curcumin molecules onto a positively charged Ru(II) complex scaffold (bipyridine for Ru1, phenanthroline for Ru2) (Figure 3K) to construct an octahedral structure, and they found that it not only inhibits the aggregation of the tau R3 region but also breaks down aggregated R3 fibers in a dose-dependent manner, while the ligand bipyridine or phenanthroline of the complex did not affect R3 aggregation. The study highlights the implications of stereochemistry, including steric hindrance effects, for the design of anti-AD drugs [107]. Massimiliano Cuccioloni et al. found that aromatic Ru(II) derivatives of curcumin (Figure 3L) not only reversibly bind to Aβ_1–40_ and Aβ_1–42_ peptides, but they also improve the curative effect of curcumin, which has a stronger hindrance to the aggregation mechanism of amyloid [108].

### 3.2. Platinum Complex

**Figure 4:**
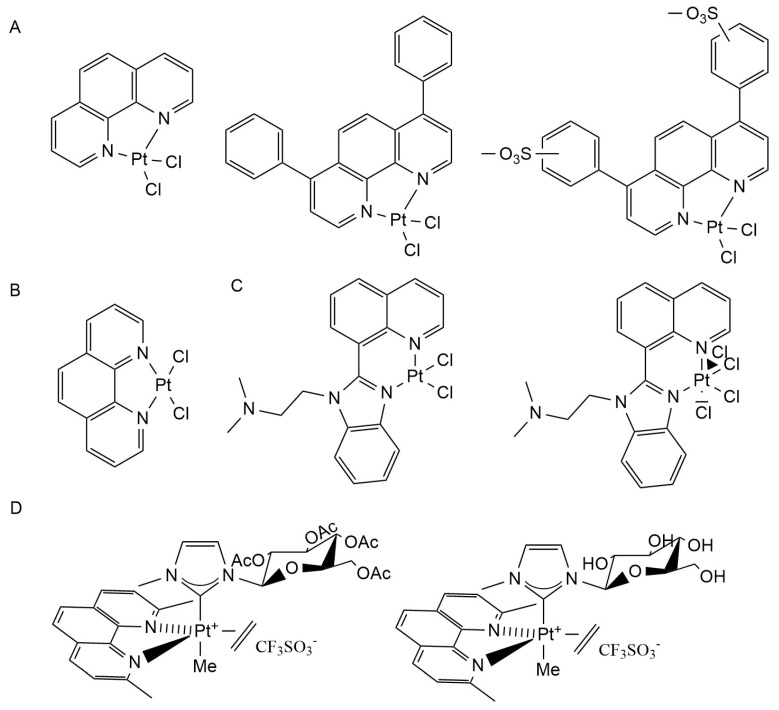
The chemical structures of platinum complexes. (**A**) Pt(1,10-phenanthroline)Cl_2_, Pt(4,7-diphenyl-1,10-phenanthroline)Cl_2_, Pt(4,7-diphenyl-1,10-phenanthroline disulfonate)Cl_2_; (**B**) platinum phenanthrol complexes(PtCl_2_(phen)); (**C**) Pt(II) and Pt(IV) complexes with 8-(1H-benzoimidazol-2-yl)-quinoline (8-BQ) as ligand; (**D**) pentacoordinate platinum(II) complexes **1Pt** and **1Pt**_dep_.

Platinum complexes mainly inhibit the aggregation of Aβ and its toxicity to neurons, in addition to blocking part of the metal ion-induced generation of reactive oxygen species. Kevin J. Barnham et al. applied metal complexes in the study of anti-Aβ aggregation for the first time. Based on the structure of the ligand 1,10-phenanthroline, they designed three Pt(II) complexes (Pt(1,10-phenathroline)Cl_2_, Pt(4,7-diphenyl-[1,10]phenanthroline)Cl_2_, Pt(4,7-diphenyl-[1,10]phenanth disulfonate)Cl_2_) (Figure 4A).The results show that the affinity of platinum-free 1,10-phenanthroline ligand to Aβ is very low, but the ligand can target histidine residues to promote the coordination of the Pt(II) complex with Aβ. L-PtCl_2_ (L = 1,10-phenanthroline derivatives) (Figure 4B) can inhibit Aβ_42_ aggregation and its neurotoxicity, including the inhibition of LTP by Aβ. In addition, L-PtCl_2_ can also block Cu^2+^-mediated reactive oxygen species generation [109]. Further studies by Guolin Ma et al. found that platinum phenanthrol complexes can most rapidly passivate Aβ to prevent its aggregation when phenanthroline ligands bind to Aβ histidine 14 and 16 [110]. To explore the role and mechanism of platinum complexes in AD animal models, Vijaya B. Kenche et al. investigated the effect of the 8-(1H-benzoimidazol-2-yl)-quinoline (8-BQ) and its Pt(II) and Pt(IV) complexes (Figure 4C) on the cytotoxicity and aggregation of Aβ in transgenic AD mouse models, suggesting that platinum complexes can prevent Aβ aggregation and plaque formation in vivo, but 8-BQ alone does not have this function [111]. Sara La Manna et al. found that two related glycoconjugate pentacoordinate Pt(II) complexes (Figure 4D) prevent Aβ aggregation by binding to Aβ_21–40_ and Aβ_25–35_ peptides at the terminal of AβC [112].

**Figure 5:**
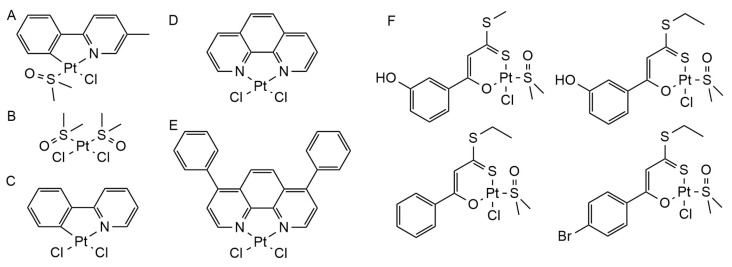
The chemical structures of platinum complexes. (**A**) [Pt(ϕ-MePy)(DMSO)Cl; (**B**) Pt(DMSO)_2_Cl_2_; (**C**) Pt(bpy)Cl_2_; (**D**) Pt(Phen)Cl_2_; (**E**) Pt(ϕ-Phen)Cl_2_; (**F**) platinum(II) complexes with different cinnamic acids.

Fabrice Collin et al. investigated five platinum(II) complex ([Pt(ϕ-MePy)(DMSO)Cl (Figure 5A), Pt(DMSO)_2_Cl_2_ (Figure 5B), Pt(bpy)Cl_2_ (Figure 5C), Pt(Phen)Cl_2_ (Figure 5D), Pt(ϕ-Phen)Cl_2_]) (DMSO = dimethyl sulfoxide, bpy = 2,2′-bipyridine) (Figure 5E) on Cu^2+^ and Zn^2+^-mediated Aβ behavior, all of whom were found to block Zn^2+^-mediated Aβ_28_ aggregation [113]. Daniele Florio et al. studied the effects of Pt(II) complexes with different cinnamic acids (Figure 5F) on NPM1_264–277_, Sup35p_7–13_, and Aβ_21–40_ amyloid proteins, and the results showed that the platinum complex could inhibit amyloid aggregation [114]. X. Wang et al. found that macrocyclic platinum chelating agents can inhibit metal-mediated Aβ aggregation and ROS production, so they constructed macrocyclic platinum chelating agents. The complex has dual properties: on the one hand, it can bind Aβ via Pt(bipyridine)Cl_2_, and on the other hand, it can trap metal ions such as copper and zinc via cycloalkene. Their study found that the macrocyclic platinum complex, in addition to having a more significant effect than the cycloalkene, can significantly inhibit Aβ aggregation and its toxicity to mouse neurons [115]. Studies by Gulshan R Walke et al. found that cisplatin inhibits Cu^2+^-mediated Aβ oxidation, and Cu^2+^-mediated ROS generation is one of the important factors leading to neurodegeneration in AD patients. Therefore, cisplatin may alleviate neuropsychiatric symptoms in AD patients by inhibiting Cu^2+^-catalyzed Aβ oxidation [116].

### 3.3. Zinc Complex

At present, few zinc complexes have been reported for the treatment of AD, mainly zinc aminocarboxylate(II) complexes, Zn_7_MT3, and pine-peptide-zinc chelates. Current studies have proved that the above complexes have antioxidant activities, and they all have their unique mechanisms of action for the treatment of AD. Their mechanisms of action will be explained in detail below.

Zn(II)-based amide carboxylate complexes are synthesized by forming stable coordination bonds with zinc ions by amide carboxylates or their derivative ligands. Wajeeha Waseem et al. found that the Zn(II)-based amide carboxylates complex has antioxidant stress activity, which can not only significantly increase the levels of superoxide dismutase (SOD), glutathione, and catalase, but it can also reduce the level of AChE and promote the release of acetylcholine neurotransmitter, which has the potential to improve behavioral disorders and change biochemical markers in the brain [117]. Rehman Zafar et al. synthesized six kinds of zinc amide carboxylate complexes ({3-[(2-methoxy-5-nitrophenyl) carbamoyl] propanoyl} zincio 3-[(2-methoxy-5-nitrophenyl) carbamoyl] propanoate(AAZ 1), (3-[(4-methoxy-2-nitrophenyl) carbamoyl] propanoyl} zincio 3-[(2-methoxy-5-nitrophenyl) carbamoyl] propanoate(AAZ 2), ({3-[(2-methoxy-5-nitrophenyl) carbamoyl] propanoyl} zincio 3-[(2-methoxy-5-nitrophenyl) carbamoyl] propanoate 2-(piperidin-2-yl) pyridine(AAZ 3),({3-[(2-methoxy-5-nitrophenyl) carbamoyl] propanoyl} zincio 3-[(2-methoxy-5-nitrophenyl) carbamoyl] propanoate 1, 10-phenanthroline(AAZ 4), ({3-[(4-methoxy-2-nitrophenyl) carbamoyl] propanoyl} zincio 3-[(2-methoxy-5-nitrophenyl) carbamoyl] propanoate 2-(piperidin-2-yl) pyridine(AAZ 5),({3-[(4-methoxy-2-nitrophenyl) carbamoyl] propanoyl} zincio 3-[(2-methoxy-5-nitrophenyl) carbamoyl] propanoate 1, 10-phenanthroline(AAZ 6 ). And they confirmed that these six complexes have good anticholinesterase inhibition and antioxidant effects. Among them, compounds AAZ 5 and AAZ 3 have better inhibitory effects on acetylcholinesterase, while AAZ 6 has been proven to be an excellent inhibitor of butyrylcholinesterase [118]. In addition, Rehman Zafar et al. synthesized two zinc amide carboxylates complexes (3-[(2-Methoxy-5-Nitrophenyl) Carbamoyl] Propanoyl Zincio 3-[(2-Methoxy-5-Nitrophenyl) Carbamoyl] Propanoate) Pyridine [Zn(L7)_2_(Pyridine)] (AAZ 7) and (3-[(4-Methoxy-2-Nitrophenyl) Carbamoyl] Propanoyl Zincio 3-[(2-Methoxy-5-Nitrophenyl) Carbamoyl] Propanoate Pyridine [Zn(L8)_2_(Pyridine)] (AAZ 8), which also showed good in vitro anticholinesterase and antioxidant activity, as well as could scour free radicals [119].

Zn_7_MT3 consists of cysteine (Cys) in metallothionein-3 (MT3) as a chelating group, and the sulfur atom (S) of Cys is combined with 7 Zn^2+^ to form a stable ring structure. MT3 is a representative zinc-binding protein that is mainly found in the brain. MT3 maintains the homeostasis of copper and zinc in cells; protects cells from oxidative stress; and regulates cell growth, differentiation, and other normal cellular functions [120]. In addition, MT3 can increase the binding of transthyroxin (TTR) to Aβ and inhibit Aβ aggregation. In the brains of AD patients, MT3 is lost [121]. W. Xu et al. injected Zn_7_MT3 into the brain of APP/PS1 mice and found that Zn_7_MT3 can significantly improve the cognitive impairment of APP/PS1 transgenic mice, improve hippocampal morphology and function, regulate metal homeostasis, eliminate Aβ plaque load, and reduce oxidative stress and neuronal apoptosis [122].

**Figure 6:**
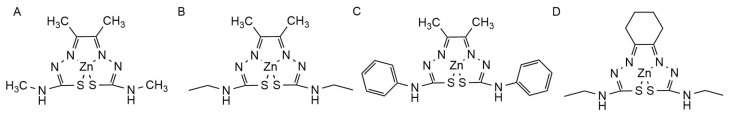
The chemical structures of ZnII[btsc]. (**A**) ZnII(atsm); (**B**) ZnII(atse); (**C**) ZnII(atsp); (**D**) ZnII(atse).

In the zinc bis(thiosemicarbazonato) complexes (ZnII[btsc]) (Figure 6A–D), Zn(II) serves as the central ion, accepting the lone electron pairs provided by the sulfur and nitrogen atoms in the thiocarbamate ligand to form a stable complex structure. In 2006, Anthony R. White and others found that there was no significant change in the level of Aβ in cells treated with metal ligand chloroquinol (CQ), but the complex ZnII(CQ)_2_ formed by CQ and zinc could promote the degradation of Aβ outside cells by upregulating matrix metalloproteinases (MMPs) [123]. However, the drawback is that ZnII(CQ)_2_ has no significant control over the release and retention of metals, so the team further designed and synthesized ZnII[btsc] and found that it not only has the function of degrading Aβ but can also transport Zn(II) into cells. Zinc, which regulates NMDA and AMPA glutamatergic receptors, as well as glycine-ionic and GABA receptors, is closely related to the balance of excitatory and inhibitory signals in the brain and is essential for memory function and behavior. However, the treatment of free-ligand btscH_2_ has no significant effect on the level of Aβ_1–40_, so it has no therapeutic potential for AD [18,124,125]. Therefore, zinc bis(thiosemicarbazone) complexes have the potential to treat AD.

In the zinc chelate of the pinecone peptide, zinc, as the central ion, interacts with some functional groups (such as carbonyl groups, carboxyl oxygen atoms, amide groups, etc.) to form a stable ring structure. Z. Zhang et al. found through in vivo experiments in AD mouse models that pine seed peptide–zinc chelates can reduce AChE level and increase choline acetyltransferase (ChAT) levels, thereby improving the functional damage of cholinergic neurons in AD, as well as also improve the levels of endogenous thiol antioxidants in the cerebral cortex, thereby playing an antioxidant role. In addition, pine peptide–zinc chelates can also play a function that Zn(II)-based amide carboxylate and Zn_7_MT3 do not have: increase lactobacillus and reduce the number of bacteroides, thus regulating the intestinal flora imbalance caused by AD [126].

Ficus acid (AA) has been reported to have many pharmacological effects, such as antioxidation, anticholinesterase, and anti-inflammation [127,128]. Wildson Max Barbosa da Silva and his team synthesized AA-Zn and AA-Cu complexes, and they confirmed for the first time that AA-Zn and AA-Cu are superior to free AA in terms of antioxidant activity; as well, AA-Cu has stronger anti-AChE activity and has the potential to treat AD [129]. Rifat Jahan et al. found that zinorthomethyl carbonoditioate (ZOMEC) can reduce oxidative stress by increasing catalase (CAT) and glutathione S transferase (GST), as well as reduce lipid peroxidation (LPO). In addition, ZOMEC can also save memory damage in AD mice and significantly inhibit Aβ accumulation, BACE1 expression, and the p-JNK pathway [130]. Abdullah Md Sheikh and Shatera Tabassum et al. found that locational methylated Zn-Phthalocyanine (Cznpc) and ZnPC (Coona) have the ability to bind with the oligomeric Aβ_1–40_ peptide and prevent subsequent fibril elongation, thus inhibiting the neurotoxic effect of Aβ [131,132].

### 3.4. Vanadium Complex

**Figure 7:**
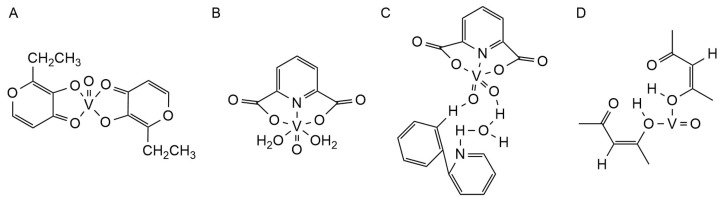
The chemical structures of vanadium complexes. (**A**) BEOV; (**B**) [VO(dipic)(H_2_O)_2_]·2H_2_O; (**C**) [VOO(dipic)](2-phepyH)·(H_2_O); (**D**) VAC.

Vanadium is a trace element that exists inside the cell as a vanadium cation (VO_2_^+^) and outside the cell as a vanadate anion (VO_3_^−^) [133]. At present, vanadium complexes are mainly used in the treatment of diabetes, cancer, and AD, among which vanadium oxides and vanadium dioxide complexes are mainly used in the treatment of AD, such as bis(ethylmaltolato) oxidovanadium(IV) (BEOV) (Figure 7A), oxyvanadium (IV), and the dioxyvanadium (V) complex dipyridine formate series [134,135,136]. In addition, it was found that these complexes have higher affordability and stability than inorganic vanadium salts [136].

Structurally, the BEOV complex contains two ethyl maltol (2-ethyl-3-hydroxy-4-pyrroic acid) ligands and one vanadium oxide. The deposition of Aβ and hyperphosphorylation of tau are currently widely recognized mechanisms of AD pathogenesis [14]. Z. He et al., through the APPSwe/PS1dE9 mouse model, found that in response to the deposition of Aβ in AD, BEOV can significantly reduce the production of Aβ by reducing the expression of β-secretase 1 in the hippocampus and cortex of AD mice, as well as promote the clearance of Aβ by enhancing the fusion of autophagolysosomes and restoring the hyperphosphorylation of the tau protein. At the same time, the recovery of autophagy flux can also promote the clearance of phosphorylated tau. In addition, Zhijun He and his team found that BEOV also reduces tau hyperphosphorylation by inhibiting protein tyrosine phosphatase-1B and regulating insulin receptor/insulin receptor substrate-1/protein kinase B/GSK3β pathways [137,138].

There are currently four dipyridinate series of vanadium(IV) and vanadium(V) complexes: (1) [VO(dipic)(H_2_O)_2_]·2H_2_O (Figure 7B), (2) [VO(dipic)(phen)]·3H_2_O, (3) [VO(dipic)(bipy)]·H_2_O, and (4) [VOO(dipic)](2-phepyH)·H_2_O (dipic=dipicolinate, phen = 1,10-phenanthroline, bipy = 2,2′-bipyridyl, 2-phephyH = 2-phenylpyridine) (Figure 7C). Structurally, the central ions of (1)–(3) are both VO_2_^+^ and have a ligand called dipicolinate, which differs in the second ligand: (1) two H_2_O, (2) 1,10- phenanthroline, (3) 2,2′-bipyridyl, and (4) are formed by the crystallization of a dioxy-(pyridine-2, 6-dicarboxylic)-vanadium(V) anion, a 2-phenylpyridine cation, and a H_2_O molecule. In the study of Joanna Drzeżdżon et al., all four complexes were shown to have antioxidant properties and the potential to treat AD, and the greater the basicity of the auxiliary ligand, the better the oxidation resistance of the complex to the superoxide anion radical. Joanna Drzeżdżon et al., for the first time, demonstrated that [VOO(dipic)](2-phepyH)·H_2_O is the strongest antioxidant, and [VO(dipic)(H_2_O)_2_]·2H_2_O is the weakest [139,140,141].

Acetylacetone oxyvanadium (VAC) (Figure 7D) is a stable complex formed by the coordination of the acetylacetone group with vanadium(V) through its oxygen atom. Yaqiong Dong and his team reported that VAC can mitigate the pathogenesis of Aβ by (1) activating the PPARγ-AMPK signal transduction pathway and improving the metabolism of glucose and energy, (2) upregulating the expression of glucose-regulated protein 75 (Grp75), reducing the level of ROS, thereby inhibiting neuronal apoptosis under p53-mediated Aβ-related stress, and (3) reducing toxic soluble Aβ peptides and alleviating neuronal damage, which has the potential to treat AD [142,143].

### 3.5. Copper Complexes

Copper is an essential trace element, and its homeostasis is related to the pathogenesis of AD. Therefore, copper complexes are considered to be potential therapeutic agents for AD, which can play an anti-AD role by anti-inflammatory, clearing Aβ, regulating metal homeostasis, etc.

**Figure 8:**
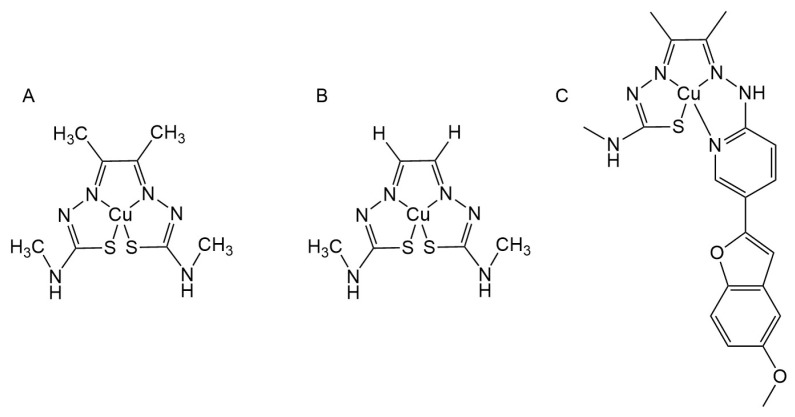
The chemical structures of copper complexes. (**A**) CuII[ATSM]; (**B**) CuII[GTSM]; (**C**) CuL5.

At present, in the treatment of AD with copper bis (thiocarbazone) complexes, copper II diacetyl bis(4-methyl-3-thiosemicarbazone) (CuII[ATSM]) (Figure 8A) and copper II glyoxal bis(4-methyl-3-thiosemicarbazone) (CuII[GTSM]) (Figure 8B) have been reported. In CuII[GTSM], the two aldehyde groups of glyoxal form coordination bonds with the amidogen (NH_2_) and thiocarbonyl (C=S) parts of the two 4-methyl-3-thiocarbazone, respectively, to connect the two thiocarbazone molecules, and they coordinate with Cu(II) to form a stable ligand structure. In ATSM, acetyl takes the place of glyoxal. Back in 2008, Paul S. Donnelly et al. found that CuII[ATSM] can release copper within cells and regulate metal homeostasis in patients with AD [125]. In addition, studies have found that P-gp can protect the brain from various endogenous and exogenous substances and has a clearing effect on Aβ, which plays an important role in brain homeostasis, and the decreased expression of P-gp in AD patients aggravates the deposition of Aβ [33,144]. Jae Pyun et al. confirmed that CuII[ATSM] can enhance the expression and function of P-gp; on the contrary, CuII[GTSM] as a substrate of P-gp efflux protein can reduce P-gp expression [145,146,147]. In addition, Xin Yi Choo and his team reported that CuII[ATSM] can induce an increase in the neuroprotective protein metallothionein-1 (MT1), the NO, monocyte chemotactic protein 1 (MCP-1), tumor necrosis factor (TNF) produced by primary microglia and NO, MCP-1, and interleukin 6 (IL-6) produced by astrocytes, which were significantly reduced, thus playing an anti-inflammatory role [148,149,150]. Therefore, CuII[ATSM] has the potential to treat AD.

A thiosemicarbazone-pyridylhydrazone Cu(II) complex (CuL5) (Figure 8C) is a thiosemicarbazone-pyridyl hydrazone containing the functional group of benzofuran. Xin Yi Choo et al. found through in vitro experiments that CuL5 has anti-inflammatory effects like CuII[ATSM], which can reduce the pro-inflammatory cytokines MCP-1 and TNF and increase the expression of MT1 [151]. In addition, microglia receptors CD33 and TREM2 have been associated with the risk of AD, and CuL5 can regulate the expression of CD33 and TREM2 in mouse microglia, as well as improve the phagocytosis function of microglia [152,153]. In addition, they treated 5xFAD mice with CuL5 in vivo and also found improvements in cognitive function in mice.

Xiaoyu Zhang et al. identified a new Cu(II) binding peptide (S-A-Q-I-A-P-H, PCu) from the heptapeptide library displayed by phages and used it as a copper ligand. Subsequently, they found that PCu could chelate Cu^2+^ and inhibit the Aβ aggregation induced by Cu^2+^ in vitro. It also attenuated Cu^2+^-mediated oxidative stress in N2a-sw cells. In addition, PCu inhibited levels of the β-secreting enzymes BACE1 and sAPPβ, thereby inhibiting the production of Aβ aggregates [154].

Valentina Oliveri and her team synthesized three new β-Cyclodextrin conjugates of 8-hydroxyquinones. These three compounds have remarkable antioxidant capacity, and they can form complexes with Cu(II) and Zn(II) in aqueous solutions, among which the Cu (II) complex shows high superoxide dismutase (SOD) activity and stronger antioxidant capacity than ligands, as well as has the potential to treat AD [155]. In addition, James L. Hickey and others have prepared a series of new tetra dentate hybrid hydroxyl quinoline-thiomicarbazone pro ligands, which have double deprotonation. As well, they can form a neutral Cu(II) complex, and it was confirmed that the complex can inactivate GSK3β, inhibit tau hyperphosphorylation, and has the potential to treat AD [156].

### 3.6. Magnesium Complex

**Figure 9:**
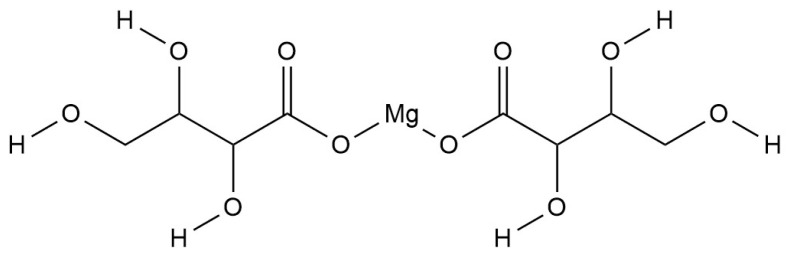
The chemical structure of MgT.

It has been reported that magnesium can accelerate toxin clearance, reduce neuroinflammation, inhibit pathological changes in amyloid precursors and tau protein hyperphosphorylation, reverse the dysregulation of NMDA receptors, and prevent nerve cells from becoming overexcited and dying [157,158,159]. In patients with AD, magnesium levels decline [160]. At present, the research on magnesium complexes for the treatment of AD has mainly focused on magnesium L-threonine (MgT) (Figure 9).

MgT plays a role in the treatment of AD through various mechanisms. First, several studies by Jun Zhang, Yanling Shen, and Wei Li et al. jointly confirmed that MgT exerts a synaptic protective effect by increasing the concentration of Mg^2+^ in the brain of a mouse model of AD, resulting in a significant increase in spatial and associative memory in mice [161,162,163,164]. Secondly, the results of Ying Huang, Xin Yu, Pu Wang, et al. showed that MgT can not only reduce the expression of inflammatory factors such as TNF-α and IL-1β but also reduce the formation of Aβ and inhibit the activation of neuroinflammation by correcting the unbalanced NMDAR signaling pathway, PI3K/Akt signaling pathway, and NF-κB-dependent mechanisms [161,165,166,167,168,169,170]. In addition, Ying Xiong and his team found that MgT can reduce the formation of ROS, downregulate the expression of Aβ_1–42_ and NADPH oxidase 4 (NOX4) proteins—among others—and resist the oxidative stress damage induced by Aβ, thus reducing the apoptosis of neurons [169]. Studies by Dinesh M Gangoda et al. have also found potential synergies between atorvastatin and MgT in enhancing memory function and reducing oxidative stress [171]. Finally, research by Wang Liao et al. found that MgT can repair intestinal barrier dysfunction and regulate the microbiome-gut-brain axis in APP/PS1 mice, reducing the secretion of gut inflammation-related factors, with potential long-term effects on neuronal activity in the brain [172].

### 3.7. Other Metal Complexes

Debo Gao et al. found that Chondroitin Li sulfate (CS-Li) can delay the development of AD through a variety of mechanisms, including inhibiting Aβ aggregation, reducing oxidative stress damage to neurons, inhibiting tau hyperphosphorylation, and controlling neuroinflammation through MAPK signaling pathways [173]. CS-Li can regulate the nuclear translocation of NF-κB by activating the MAPK signaling pathway to reduce the expression of pro-inflammatory factors.

**Figure 10:**
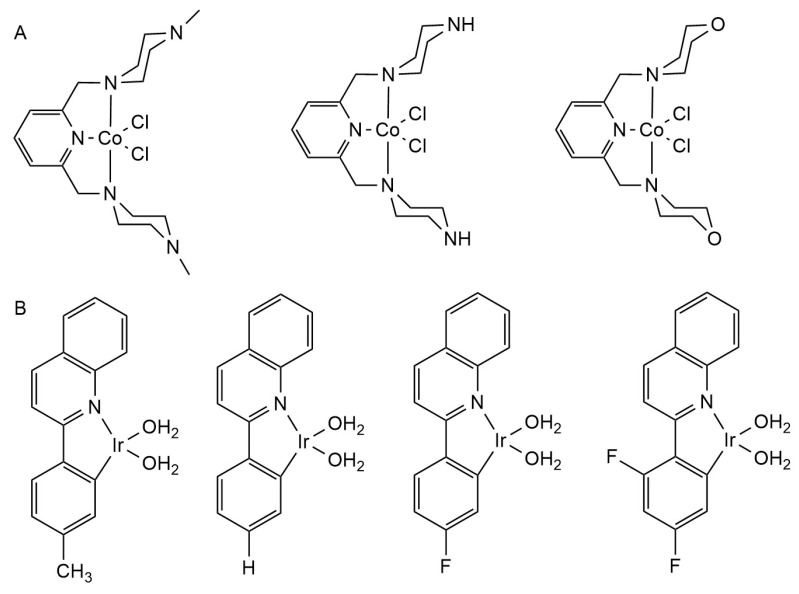
The chemical structures of other complexes. (**A**) NNN-L1, NNN-L2, NNN-L3; (**B**) IR-Me, IR-H, IR-F.

Nalini Vijay Gorantla et al. identified 2,6-bis (4-methylpiperazine-1-yl-methyl) Pyridine (NNN-L1), 2,6-bis (piperazin-1-yl-methyl) Pyridine Three ligands (NNN-L2), and 2,6-bis (morpholinomethyl) Pyridine (NNN-L3), which were combined with cobalt chloride to synthesize (NNN-L1) CoCl_2_, (NNN-L2) CoCl_2_ and (NNN-L3) CoCl_2_ (Figure 10A). These complexes can effectively inhibit tau aggregation and decompose the formed tau fibers; they also have the potential to treat AD [174].

Juhye Kang et al. designed four Ir(III) complexes (IR-Me, IR-H, IR-F, and IR-F_2_) (Figure 10B) that can be covalently bound to Aβ, replacing the two H_2_O molecules bound to the center of Ir(III) into Aβ without the influence of light and O_2_ [175]. In the presence of light and oxygen, Ir(III) complexes bound to Aβ induce intramolecular and intermolecular oxidation of Aβ at histidine 13, histidine14, and/or methionine 35. By modifying the Aβ peptide through coordination and oxidation, Aβ aggregation and cytotoxicity can be effectively controlled to treat AD.

## 4. Outlook and Perspective

In summary, metal complexes can treat AD through various mechanisms, such as reducing the formation and aggregation of Aβ and tau, anti-oxidative stress, regulating intestinal flora, etc. They have great potential in the treatment of AD. However, drugs present a major challenge in the treatment of brain diseases—the presence of the BBB—so developing effective transport and delivery systems is critical. Currently, peptide polylactic glycolic acid (PLGA) nanoparticles have been reported to be loaded with MasR agonist PNA5 to treat AD across the BBB, demonstrating the potential for precise drug delivery using nanoparticles [176]. There is currently a lot of research devoted to developing Aβ antibodies, and the innovation of the Aβ monoclonal antibody Trontinemab is its human transferrin 1 (TfR1)-oriented BrainshuttlTM module; thus, circulatory trontinemab can bind to transferrin receptors on endothelial cells that form the BBB, thereby using TfR1-mediated endocytosis to deliver drugs into the brains of non-human primates. Therefore, binding metal complexes to structures targeting TfR1 may hold promise for precise intervention in the pathological process of AD [177].

At the same time, there are shortcomings in the safety, stability, and clinical trials of metal complexes in the treatment of AD. Many studies have shown that the change in metal homeostasis is an important factor in the pathogenesis of AD. Excessive plasma amounts of iron, copper, zinc, and aluminum can lead to neurotoxicity. Therefore, the dosage of metal complexes should be controlled within a safe range to prevent further damage to neurons [18,19,20,178,179,180]. The stability of metal complexes depends on a variety of factors, such as crystal field stabilization energy (CFSE), effective atomic number to obtain chelation degree of ligand, pH, etc., so it is also necessary to improve the stability of metal complexes to ensure their effectiveness and durability in vivo [181]. At present, although metal complexes for the treatment of AD have made many advances in laboratory studies, there are few clinical trials, which has also led to the limitations regarding their safety studies.

## Abbreviations

The following abbreviations are used in this manuscript:

5-HT	5-Hydroxytryptamine
AChE	Acetylcholinesterase
AD	Alzheimer’s disease
AMPA	Alpha-amino-3-hydroxy-5-methyl-4-isoxazolepropionic acid
APOE ϵ 4	Apolipoprotein ϵ 4
APP	Amyloid precursor protein
BACE1	β-secretase
BBB	Blood–brain barrier
BEOV	Bis(ethylmaltolato) oxidovanadium(IV)
BODIPY	Boron-dipyrromethene
BuChE	Butyrylcholinesterase
CA	Catecholamine
CDK5	Cyclin-dependent kinase 5
CFSE	Crystal field stabilization energy
ChAT	Choline acetyl transferase
CQ	Clioquinol
CS-Li	Chondroitin Li sulfate
Cys	Cysteine
DA	Dopamine
DMT	Disease-modifying therapy
FDA	The U.S. Food and Drug Administration
GABA	Gamma-aminobutyric acid
Grp75	Glucose-regulated protein 75
GSH	Glutathione
GSK3β	Glycogen synthase kinase 3 β
IAPP	Islet amyloid polypeptide
IL-6	Interleukin 6
LC	Locus coeruleus
LD	Linear dichroism
LRP	Lipoprotein receptor-associated protein
LTD	Long-term depression
LTP	Long-term potentiation
MCP-1	Monocyte chemotactic protein 1
MGnD	Microglial neurodegenerative phenotype
MgT	Magnesium L-threonine
MMP	Matrix metalloprotease
MT	Metallothionein
NA	Norepinephrine
NAcc	Nucleus accumbens
NBM	Nucleus basalis of Meynert
NFT	Neurofibrillary tangle
NMDA	N-Methyl-D-aspartic acid
NO	Nitric oxide
NOX4	NADPH oxidase 4
p38 MAPK	p38 mitogen-activated protein kinase
PAM	Morphologically activated microglia
P-gp/ABCB1	P-glycoprotein
PLGA	Polylactic glycolic acid
PSEN	Presenilin
RAGE	Receptor of advanced glycation end product
ROS	Reactive oxygen species
sAPPα	soluble APPα fragment
SAR	Structure–activity relationship
SELEX	Systematic evolution of ligands by exponential enrichment
SOD	Superoxide dismutase
TfR1	Transferrin 1
TNF	Tumor necrosis factor
TTR	Transthyroxin
VAC	Acetylacetone oxyvanadium
Vps10	Vacuolar protein sorting 10
